# Patterns and determinants of premature mortality from non-communicable diseases in Aktobe region, Kazakhstan: a mixed-methods study

**DOI:** 10.3389/fpubh.2026.1813999

**Published:** 2026-06-15

**Authors:** Dinara Katasheva, Lyazzat Kosherbayeva, Talgar Abilov, Nurgul Alekenova, Lorena Dini

**Affiliations:** 1 West-Kazakhstan Medical University, Aktobe, Kazakhstan; 2 Asfendiyarov Kazakh National Medical University, Almaty, Kazakhstan; 3 Al-Farabi Kazakh National University, Almaty, Kazakhstan; 4 Charité – Universitätsmedizin Berlin, Berlin, Germany

**Keywords:** health policy, Kazakhstan, non-communicable disease, premature mortality, public health

## Abstract

**Background:**

Non-communicable diseases (NCDs) cause approximately 17 million premature deaths annually and are largely associated with modifiable risk factors.

**Methods:**

This sequential explanatory mixed-methods study combined descriptive trend analysis of premature NCD mortality with qualitative inquiry. Quantitative data from the National Scientific Center for Health Development (Aktobe region, 2019–2023) were analyzed using Joinpoint Regression and Statistical Package for the Social Sciences (SPSS) statistical software packages. Premature mortality was defined as death before age 75, and crude (non-age-standardized) rates per 100,000 population were calculated. Trends were summarized using average annual percentage change (AAPC) with 95% confidence intervals (CIs). Semi-structured interviews with 10 healthcare managers explored awareness of premature mortality indicators, perceived risk factors, and the use of data in decision-making.

**Results:**

Premature mortality fluctuated over the study period, peaking in 2021 and declining by 2023 (AAPC –4.9%; 95% CI: −18.8 to 11.3), indicating no statistically significant overall trend. Cardiovascular diseases (CVDs) and cancer were the leading causes, with consistently higher rates among men and urban populations. Statistically significant declines were observed for cancer and diabetes in some subgroups, while other trends, including respiratory mortality, were not statistically significant and showed substantial variability. Qualitative findings highlighted gaps in the use of epidemiological data for strategic decision-making and identified system-level barriers to effective data utilization.

**Conclusion:**

Premature mortality from NCDs remains substantial in the Aktobe region, with persistent gender and urban–rural disparities. While these findings provide context-specific insights for regional health planning, they should be interpreted with caution given the descriptive design, the use of crude rates, and the short observation period. Strengthening data-driven decision-making and preventive strategies may help address observed disparities, but further analytical and longitudinal research is needed to better understand the underlying drivers.

## Introduction

Non-communicable diseases (NCDs) remain a leading cause of premature mortality globally and are projected to increase further due to population aging (1). According to the World Health Organization, approximately 17 million people die each year from NCDs before the age of 70, with the majority occurring in low- and middle-income countries (2). Cardiovascular diseases (CVDs), cancer, chronic respiratory diseases, and diabetes are the primary contributors (2, 3), and these conditions are largely associated with modifiable behavioral and metabolic risk factors (3). This highlights the critical role of prevention, early detection, and effective health system interventions in reducing premature mortality (4).

Global efforts to reduce NCD-related premature mortality have focused on strengthening primary healthcare (PHC), improving ` to screening and treatment, and addressing social determinants of health (4). While these strategies have demonstrated effectiveness in many settings, outcomes vary substantially depending on local health system capacity, data utilization, and regional socioeconomic conditions (5). In transitional and middle-income countries, health system performance is often constrained by limited integration of epidemiological data into decision-making processes and insufficient coordination between preventive and clinical services (5).

Kazakhstan has made substantial progress in strengthening its PHC system, including increased financing, implementation of disease management programs, and expansion of screening initiatives (6–9). Despite these efforts, significant disparities persist, particularly in life expectancy between men and women, and between urban and rural populations (10). Existing research has largely focused on national-level indicators of avoidable mortality, with limited attention to regional variations and the health system factors that may influence these patterns (11).

Aktobe Region represents an important setting for examining premature mortality in Kazakhstan due to its unique demographic, geographic, and socioeconomic characteristics. The region is one of the largest industrial areas in western Kazakhstan, with substantial mining, oil, gas, and metallurgical activities that may contribute to environmental and occupational health risks. In addition, Aktobe Region includes both densely populated urban centers and geographically remote rural settlements, creating significant disparities in healthcare accessibility and service utilization (12). Compared with several other regions of Kazakhstan, Aktobe has demonstrated persistent differences in mortality indicators, workforce shortages in rural healthcare facilities, and varying levels of preventive healthcare coverage. These characteristics make the region particularly relevant for exploring premature mortality patterns and the practical use of epidemiological indicators in regional health system management.

Moreover, while quantitative indicators such as premature mortality rates are increasingly used to evaluate health system performance (5), there is limited understanding of how healthcare managers interpret and implement by these indicators in practice. Previous studies have rarely combined epidemiological analysis with qualitative insights from health system stakeholders, creating a gap between statistical evidence and real-world decision-making processes.

This study is guided by a health systems framework in which premature mortality is conceptualized as the result of interactions among population-level risk factors, healthcare access, and health system responsiveness (10). Within this framework, quantitative analysis is used to identify patterns and disparities in premature mortality, while qualitative inquiry explores how these patterns are understood and utilized within the health system. This approach allows for a more comprehensive interpretation of premature mortality by linking epidemiological trends with system-level processes.

Against this background, this study addresses two key gaps. First, it provides a regional analysis of premature mortality from NCDs in Aktobe Region, disaggregated by gender and urban–rural residence, thereby extending existing national-level evidence. Second, it examines healthcare managers’ perspectives on the use of premature mortality indicators, including their awareness of, application, and perceived barriers to data use. By integrating quantitative and qualitative approaches, this study offers a more comprehensive understanding of premature mortality and helps bridge the gap between epidemiological evidence and health system decision-making in Kazakhstan.

## Methods

### Study design

This study employed a sequential explanatory mixed-methods design, where qualitative findings were used to complement and interpret quantitative results. The quantitative and qualitative components were conducted sequentially and integrated at the interpretation stage to provide a comprehensive understanding of premature mortality patterns and their use in health system decision-making. Integration of quantitative and qualitative findings was conducted using a narrative approach at the interpretation stage. Integration was guided by a triangulation approach in line with recognized mixed-methods integration strategies, focusing on convergence, complementarity, and explanation. Qualitative findings were used to explain observed quantitative patterns, particularly differences across population groups and trends over time.

### Quantitative data and measures

Mortality data were obtained from the National Scientific Center for Health Development (Aktobe Region) for the period 2019–2023. The dataset included all registered deaths disaggregated by age, sex, place of residence (urban/rural), and cause of death according to the *International Classification of Diseases, 10th Revision* (ICD-10).

NCDs included cardiovascular diseases, cancer, diabetes, respiratory diseases, and other NCD categories (detailed ICD-10 classification is provided in Supplementary material 1). The respiratory disease category (ICD-10 codes J00–J99) includes both chronic and acute conditions, including infectious diseases such as influenza and pneumonia. Therefore, this category does not exclusively represent non-communicable diseases and should be interpreted with caution. Age groups were categorized in five-year intervals (0–4, 5–9, …, 70–74 years).

Premature mortality was defined as death before age 75 (13). All rates are crude (not age-standardized). Accordingly, mortality rates were calculated using the population under age 75 as the denominator:

Number of deaths before age 75/Population under age 75 × 100,000.

Premature mortality was expressed as crude rates per 100,000 people. The analysis aims to provide a descriptive overview of premature mortality patterns using routinely collected administrative data. Crude rates were used to reflect the actual observed burden within the population and to maintain transparency for health system stakeholders. Adjustment for confounders and age-standardization were not performed due to the descriptive nature of the analysis and the limited availability of detailed covariate data. The use of crude rates means that observed differences between groups may partly reflect variations in underlying age structures rather than true differences in mortality risk.

Absolute differences and relative changes (%) were calculated by sex, residence, and time period. Trends were assessed using average annual percentage change (AAPC) with 95% confidence intervals.

AAPC values summarize the average trend over the study period. When 95% confidence intervals include zero, the trend should be interpreted as statistically non-significant. Wide confidence intervals reflect uncertainty in trend estimates and may result from small numbers of events or limited time points.

Analyses were conducted using the Joinpoint Regression Program (version 4.9.1.0) (14), Microsoft Excel, and Statistical Package for the Social Sciences (SPSS) version 13.0. All estimates are unadjusted descriptive rates derived from population-level administrative data and were not adjusted for age or other potential confounders. Given the limited number of time points (2019–2023), AAPC estimates were used descriptively to summarize trends and should be interpreted cautiously. Differences between groups should be interpreted with caution due to potential variations in age structure.

### Qualitative study design and participants

The qualitative component used an interpretive descriptive approach—commonly used in health systems and applied health research to explore professional experiences, perceptions, and decision-making processes in real-world clinical and administrative settings. This approach was chosen because the study aimed not only to describe premature mortality trends quantitatively but also to understand how healthcare managers interpret and utilize mortality indicators in practice.

Semi-structured interviews were conducted with healthcare managers working in regional medical organizations in Aktobe Region. A purposive sampling strategy was employed to recruit participants occupying key managerial and administrative positions within the regional health system. Participants were selected for their involvement in health planning, screening programs, epidemiological monitoring, or the implementation of preventive services, ensuring the inclusion of individuals directly engaged in decision-making related to non-communicable disease prevention and population health management.

A total of 20 healthcare managers were invited via official email; 10 agreed to participate (response rate: 50%). Although participation was voluntary and may introduce selection bias, participants represented a range of managerial roles and healthcare settings across the region, enabling exploration of diverse perspectives on premature mortality and data utilization.

### Data collection

Interviews were conducted between January and June 2025 and lasted approximately 30–45 min. A semi-structured interview guide was developed based on the study’s objectives, the health systems framework underpinning it, and existing literature on mortality surveillance, preventive health services, and health system performance.

The semi-structured format allowed researchers to explore predefined topics while also enabling participants to elaborate on issues they considered important within their professional experience. Interview topics included: awareness and understanding of premature mortality indicators; perceived regional risk factors contributing to premature mortality; monitoring and use of epidemiological data; barriers to effective data utilization; intersectoral collaboration; and the role of healthcare managers in reducing premature mortality.

All interviews were audio-recorded with participants’ informed consent, transcribed verbatim, and anonymized prior to analysis to ensure confidentiality.

### Qualitative analysis

Qualitative data were analyzed using thematic content analysis through an inductive, iterative process. Initial open coding was conducted independently by two researchers to identify recurring concepts, experiences, and perceptions from the interview transcripts. Codes were subsequently compared, discussed, and grouped into broader thematic categories through iterative review and consensus building.

Themes were developed inductively from the data rather than being predefined, allowing findings to reflect participants’ perspectives and experiences. Throughout the analysis, transcripts were repeatedly reviewed to ensure consistency between coded themes and original participant narratives.

To enhance methodological rigor and trustworthiness, several strategies were employed, including investigator triangulation, independent coding review, iterative refinement of thematic categories, and regular discussion among the research team to resolve discrepancies and minimize interpretive bias. Data collection continued until thematic saturation was reached, defined as the point at which additional interviews did not generate substantially new themes or interpretations.

Qualitative findings were integrated with quantitative results during the interpretation stage using a triangulation approach to provide contextual understanding of observed mortality patterns and disparities. Data were managed and coded manually, and no qualitative analysis software was used.

### Statistical considerations

Missing data in the mortality dataset were minimal; therefore, no imputation procedures were performed. Analyses were descriptive and trend-based, and no causal inference was attempted. Since population-level administrative data were used, a sample size calculation was not applicable. Adjustment for multiple comparisons was not performed, and findings should be interpreted as exploratory. Given the short observation period (2019–2023), trend estimates should be interpreted cautiously, particularly where confidence intervals were wide. Formal robustness testing was limited by the short study period and the dataset’s aggregate, administrative nature. However, several analytical precautions were taken, including stratified analyses by sex and residence, cautious interpretation of non-significant AAPC estimates, and explicit acknowledgment of potential reporting biases during the coronavirus disease 2019 (COVID-19) period. The study design and data sources may be subject to reporting inaccuracies and selection bias; these issues are discussed further in the “Limitations” section.

### Ethical approval

The study was approved by the Local Bioethics Committee of the West-Kazakhstan Medical University, Aktobe, Kazakhstan (№09–09–01-03, September 29, 2024), Aktobe, Kazakhstan.

## Results

### Overall premature mortality

Premature mortality increased from 512.9 per 100,000 population in 2019 to a peak of 632.7 in 2021, followed by a decline to 453.2 in 2023 (Table 1). The average annual percentage change (AAPC) was −4.9% (95% CI: −18.8 to 11.3), indicating no statistically significant overall trend.

**Table 1:** Premature mortality from non-communicable disease in Aktobe Region between 2019 and 2023, by urban and rural area and gender.

Total				
Year	Death	Total	Men	Women
2019	2,952	512.9	762.8	321,8
2020	3,394	580.3	860.7	364,4
2021	3,762	632.7	920.1	410,6
2022	2,848	451.3	694.6	259,5
2023	2,961	453.2	666.5	282,6
AAPC		−4,9 (−18,8; 11,3)	−4.9 (−17.3; 9.4)	−5.7 (−22.6; 15.0)
Urban				
2019	2,211	546.4	827.5	340,9
2020	2,517	606.6	910.1	383,7
2021	2,884	680.8	1016.6	434,2
2022	2,102	451.6	711.8	256,2
2023	2,269	467.8	701.2	289,1
AAPC		−5.1 (−21.3; 14.6)	3.3 (−14.0; 24.1)	−0,3 (−21,9; 27,2)
Rural				
2019	741	432.3	619.9	273,0
2020	877	516.7	752.9	313,4
2021	878	510.3	695.9	345,1
2022	746	449.4	647.4	270,0
2023	692	410.9	577.6	262,4
AAPC		−4,5 (−31,1; 32,3)	3.4 (−40.5; 79.7)	−8.0 (−43.4; 49.4)

As this study is based on population-level administrative data, baseline comparability between groups is descriptive and reflects underlying population differences rather than controlled group allocation. AAPC, annual average percentage changes.

Premature mortality was consistently more than twice as high among men compared to women, indicating a substantial and persistent gender disparity. Mortality rates were also higher among urban residents compared to rural populations (Table 1). Although the urban–rural gap narrowed in the final years of the study period, differences remained notable, indicating persistent inequalities in premature mortality across population groups.

### Premature mortality due to circulatory diseases

Premature mortality from circulatory system diseases decreased slightly from 270.1 to 253.7 per 100,000 population over the study period, with a notable peak in 2021 (397.2) (Table 2). The AAPC was −2.6% (95% CI: −23.6 to 24.0), suggesting no statistically significant change.

**Table 2:** Premature mortality by each non-communicable disease between 2019 and 2023 in Aktobe Region by urban and rural area and gender.

Year	Diseases of the circulatory system				Cancer				Diseases of the respiratory system			
	Death	Rate per 100,000 (total)	Men	Women	Death	Rate per 100,000 (total)	Men	Women	Death	Rate per 100,000 (total)	Men	Women
2019	1,568	270.1	442.7	133.8	912	157.2	189.3	135.3	365	66.4	106.1	37.6
2020	1,702	288.1	461.9	150.5	840	141.8	187.0	108.4	758	134.4	192.4	91.8
2021	2,350	397.2	615.2	225.7	853	139.7	141.0	112.7	461	79.4	114.6	52.7
2022	1,624	255.0	417.2	123.2	784	124.2	159.4	99.3	378	62.1	102.5	31.4
2023	1,661	253.7	414.4	122.6	830	128.1	151.9	110.3	411	62.7	92.1	40.5
AAPC		−2.6 (−23.6; 24.0)	−2.5 (−20.7; 19.8)	−3.8 (−31.9; 35.9)		−5.3* (−9.1; −1.3)	−5.9 (−14.5; 3.7)	−5.0 (−13.4; 4.2)		−12.2 (−40.5; 29.7)	−11.6 (−36.5; 23.1)	−13.8 (−48.2; 43.6)
Urban												
2019	1,208	296.6	492.5	149.6	672	164.8	200.5	140.8	246	63.4	105.4	34.4
2020	1,286	309.2	500.2	165.8	608	143.2	189.8	110.6	548	135.8	194.9	93.8
2021	1,850	440.7	702.4	245.8	650	148.3	193.3	117.8	308	73.8	106.6	49.8
2022	1,242	265.2	441.5	129.3	564	120.9	159.3	94.7	254	56.0	95.5	27.2
2023	1,295	267.5	453.0	123.2	638	132.4	153.3	117.7	298	60.4	87.7	40.4
AAPC		−6.9 (−26.7; 18.2)	1.6 (−20.4; 29.5)	3.1 (−27.7; 46.9)		−6.3*(−12.1; −0.2)	0.7 (−12.6; 16.1)	3.2 (−11.6; 20.4)		8.9 (−33.7; 79.0)	17.0 (−11.6; 54.8)	−16.7 (−45.4; 27.0)
Rural												
2019	360	207.0	333.5	93.0	240	138.9	164.3	121.8	119	73.3	107.2	45.7
2020	416	236.1	376.0	110.7	232	139.0	182.6	102.3	210	131.4	188.0	86.5
2021	500	285.9	412.3	169.6	203	118.2	141.9	98.3	152	93.4	133.7	60.7
2022	382	224.6	352.0	103.6	220	133.4	158.4	114.2	124	79.7	120.6	44.5
2023	366	213.4	314.1	120.8	192	115.6	148.5	87.4	113	69.9	104.9	40.6
AAPC		−1.8 (−23.9; 26.8)	4.7 (−39.0; 79.7)	−3.7 (−49.5; 83.7)		−6.0 (−37.2; 40.6)	3.7 (−37.5; 71.8)	−11.2 (−43.9; 40.4)		−10.6 (−43.7; 41.8)	−1.7 (−53.4; 107.4)	−12.2 (−42.1; 33.2)

Year	Endocrine, nutritional, and metabolic diseases total				Diabetes				Other disease			
	Death	Rate per 100,000 (Total)	Men	Women	Death	Rate per 100,000 (Total)	Men	Women	Death	Rate per 100,000 (Total)	Men	Women
2019	70	13.2	17.9	9.9	66	12.6	17.6	9.1	37	5.9	6.8	5.2
2020	74	13.1	16.8	10.4	67	12.0	15.7	9.2	20	2.9	2.6	3.3
2021	78	13.5	10.6	15.6	71	12.7	10.6	14.1	20	2.9	1.7	3.8
2022	42	7.1	10.6	4.4	40	6.9	10.1	4.4	20	2.9	4.9	1.2
2023	41	6.1	6.3	6.0	38	5.7	5.8	5.7	18	2.6	1.8	3.1
AAPC		−18.0(−35.6; 4.3)	−21.8* (−32.6; −9.3)	−13.2(−48.8; 47.3)		−17.8 (−34.6; 3.2)	−22.3* (−32.0; −11.1)	−12.4 (−46.4; 43.2)		−18.2 (−35.6; 4.0)	−18.4 (−52.3; 39.6)	−14.9 (−41.4; 23.8)
Urban												
2019	54	14.5	20.4	10.4	51	13.9	19.9	9.6	31	7.1	8.8	5.8
2020	60	15.2	23.0	9.7	56	14.2	21.7	8.8	15	3.1	2.3	3.8
2021	63	15.4	12.9	17.2	58	14.6	12.9	15.7	13	2.5	1.4	3.5
2022	29	6.7	10.1	4.2	28	6.5	9.6	4.2	13	2.8	5.4	0.7
2023	26	5.3	6.2	4.7	23	4.8	5.5	4.3	12	2.3	1.1	3.1
AAPC		−11.8(−45.3; 42.1)	10.3 (−33.7; 83.6)	6.6 (−46.3; 111.4)		−21.1 (−38.2; 0.7)	−22.0 (−43.4; 7.7)	−12.8 (−57.8; 80.6)		- 20.7(−46.6; 17.7)	−31.7(−67.7; −1.6)	−1.4 (−44.4; 74.9)
Rural												
2019	16	10.0	12.3	8.6	15	9.5	12.3	7.7	6	3.1	2.6	3.9
2020	14	7.8	2.9	12.1	11	6.4	2.3	10.0	5	2.5	3.5	1.8
2021	78	47.8	5.4	11.3	13	7.9	5.4	9.7	7	3.9	2.6	5.1
2022	13	8.3	12.0	5.1	12	8.1	11.5	5.1	7	3.3	4.2	2.7
2023	15	8.6	6.8	10.2	15	8.6	6.8	10.2	6	3.4	3.4	3.4
AAPC		−4.2(−34.6; 40.5)	6.3 (−15.3; 33.4)	−9.5 (−41.0; 38.9)		−1.5 (−34.9; 49.0)	5.7 (−19.1; 38.3)	−6.4 (−37.1; 39.3)		4.2 (−21.1; 37.5)	13.3 (−29.4; 81.7)	−10.1 (−58.1; 93.1)

Mortality rates are expressed per 100,000 population under age 75. AAPC is reported with 95% confidence intervals. *Statistically significant trends. All estimates are crude and not adjusted for age or other confounders. AAPC, average annual percentage change.

Mortality rates were consistently higher in urban areas compared to rural areas and approximately three times higher among men than women, reflecting substantial and persistent disparities. An increase in women mortality in rural areas was observed (93.0 to 120.8 per 100,000); however, this change was not statistically significant and should be interpreted with caution.

### Premature mortality due to cancer

Premature mortality from cancer showed a significant decreasing trend, with an AAPC of −5.3% (95% CI: −9.1 to −1.3) (Table 2). In urban populations, mortality declined from 164.8 to 120.9 between 2019 and 2022, followed by a slight increase in 2023 (132.4), with an AAPC of −6.3% (95% CI: −12.1 to −0.2) (Table 2).

In contrast, cancer mortality in rural areas fluctuated without a statistically significant trend (AAPC –6.0%; 95% CI: −37.2 to 40.6) (Table 2). Mortality remained consistently higher among men, although both sexes showed overall reductions. These findings indicate a statistically significant reduction in cancer-related premature mortality, particularly in urban settings.

### Premature mortality due to respiratory diseases

Premature mortality from respiratory diseases fluctuated over time, ranging from 66.4 to 62.7 per 100,000 population, with a peak in 2020 (134.4) (Table 2). The AAPC was −12.2% (95% CI: −40.5 to 29.7), indicating no statistically significant trend.

Mortality rates were more than twice as high among men compared to women and were generally higher in rural populations than urban populations (Table 2). These differences suggest potential disparities related to environmental exposures or access to healthcare; however, given the absence of statistically significant trends, these patterns should be interpreted with caution.

### Premature mortality due to endocrine and metabolic diseases

Premature mortality from endocrine, nutritional, and metabolic diseases declined from 13.2 to 6.1 per 100,000 population (AAPC –18.0%; 95% CI: −35.6 to 4.3), although this trend was not statistically significant (Table 2). A statistically significant reduction was observed among men (AAPC –21.8%; 95% CI: −32.6 to −9.3), while trends among women were not statistically significant. This may indicate potential improvements in disease management among males; however, overall findings should be interpreted with caution, given the absence of a statistically significant trend.

### Diabetes-specific mortality

Premature mortality due to diabetes declined from 12.6 to 5.7 per 100,000 population (AAPC –17.8%; 95% CI: −34.6 to 3.2) (Table 2). Among men, the reduction was significant (AAPC –22.3%; 95% CI: −32.0 to −11.1), particularly between 2022 and 2023 (Table 2). Urban populations showed greater reductions compared to rural populations, where mortality remained relatively stable. These differences may indicate variations in access to care or disease management; however, given the descriptive design, short observation period, and non-significant overall trend, causal or definitive interpretation is not warranted.

### Other NCDs

Premature mortality from other NCDs decreased from 5.9 to 2.6 per 100,000 population (AAPC –18.2%; 95% CI: −35.6 to 4.0) (Table 2). Mortality was higher among men during majority of the study period, although this pattern reversed in 2023. Given the small numbers and wide confidence intervals, these findings should be interpreted with caution.

### Absolute and relative changes

The largest absolute reductions were observed for cancer (−24.95 per 100,000) and circulatory diseases (−11.19 per 100,000), indicating that these conditions contributed most to the overall decline in premature mortality (Table 3). Relative reductions were greatest for endocrine and metabolic diseases (−0.39), suggesting substantial proportional improvement despite lower absolute burden.

**Table 3:** Absolute and relative changes of premature mortality by each non-communicable disease between 2019 and 2023 in Aktobe Region by urban and rural area and gender.

	Absolute change per 100,000 population			Relative change (%)		
	Men	Women	Total	Men	Women	Total
Total						
Cancer	−29.11	−37.40	−24.95	−0.19	−0.20	−0.18
Endocrine, nutritional, and metabolic diseases	−7.13	−11.62	−3.93	−0.54	−0.65	−0.39
Diabetes	−6.90	−11.80	−3.40	−0.55	−0.67	−0.37
Diseases of the circulatory system	−16.45	−28.32	−11.19	−0.06	−0.06	−0.08
Diseases of the respiratory system	−3.65	−14.05	2.90	−0.05	−0.13	0.08
NCD others	−3.38	−4.99	−2.07	−0.57	−0.74	−0.40
Total	−59.72	−96.38	−39.24	−0.12	−0.13	−0.12
Urban						
Cancer	−32.38	−47.25	−23.12	−0.20	−0.24	−0.16
Endocrine, nutritional and metabolic diseases	−9.24	−14.20	−5.69	−0.64	−0.70	−0.55
Diabetes	−9.10	−14.40	−5.30	−0.65	−0.72	−0.55
Diseases of the circulatory system	−29.16	−39.52	−26.42	−0.10	−0.08	−0.18
Diseases of the respiratory system	−2.98	−17.66	6.09	−0.05	−0.17	0.18
NCD others	−4.89	−7.68	−2.68	−0.68	−0.88	−0.46
Total	−78.66	−126.30	−51.81	−0.14	−0.15	−0.15
Rural						
Cancer	−23.31	−15.87	−34.43	−0.17	−0.10	−0.28
Endocrine, nutritional and metabolic diseases	−1.32	−5.47	1.56	−0.13	−0.45	0.18
Diabetes	−0.90	−5.50	2.50	−0.09	−0.45	0.32
Diseases of the circulatory system	6.43	−19.48	27.78	0.03	−0.06	0.30
Diseases of the respiratory system	−3.42	−2.31	−5.05	−0.05	−0.02	−0.11
NCD others	0.26	0.82	−0.48	0.08	0.32	−0.12
Total	−21.36	−42.30	−10.62	−0.05	−0.07	−0.04

Absolute change represents the difference in mortality rates (per 100,000 population) between 2019 and 2023. Relative change (%) represents proportional change over the same period. Negative values indicate reductions; positive values indicate increases.

### Qualitative findings

Key qualitative findings related to healthcare managers’ perspectives on premature mortality are summarized in Table 4. Qualitative data were analyzed using thematic coding with iterative category refinement until thematic saturation was reached. Analytical rigor was strengthened through independent coding review and consensus discussion among the research team. The majority of the participants were familiar with the concept of premature mortality, although its practical application varied. Behavioral risk factors and chronic conditions such as cardiovascular diseases, diabetes, and obesity were consistently identified as key contributors.

**Table 4:** Summary of key findings and recommendations on premature mortality reduction.

Topic	Key findings	Recommendations
Familiarity with premature mortality	Majority of healthcare managers know the concept; some apply it in practice, others less so.	Increase practical training and integration of premature mortality indicators at the PHC level to ensure consistent use.
Main medical risk factors	Harmful habits and chronic diseases (CVD, diabetes, and obesity) are the main risks.	Focus prevention efforts on modifying harmful habits, early detection, and management of chronic diseases; strengthen cardiovascular and cancer screening programs.
Risk monitoring systems	Use of age-based screenings, health schools, and education campaigns.	Expand and promote health screening programs and awareness campaigns; integrate risk monitoring into routine care.
Data collection and use	Screening data guides follow-up by multidisciplinary teams; national stats aid decisions.	Enhance data systems to enable timely collection, analysis, and sharing; support multidisciplinary follow-up models; leverage national statistics for strategic planning.
Barriers and collaboration	Barriers: funding, staff shortages, low compliance, and low health responsibility; Collaboration: through info/material exchange.	Address resource gaps; boost patient engagement; enhance partnerships.
Future role of healthcare managers	Expected to lead quality control, mortality reduction programs, data-driven decisions, and prevention efforts. Support leadership and integrated data use.	Empower healthcare managers with training and resources; promote leadership roles in quality and prevention; support development of integrated data and decision-support systems.

Table summarizes themes identified through thematic analysis of semi-structured interviews with healthcare managers (*n* = 10).

Participants reported that data from screening programs are used for patient monitoring, although their use in strategic decision-making remains limited. Key barriers included financial constraints, workforce shortages, and low patient compliance. One participant noted, “We collect a lot of data, but it is rarely used for planning decisions.” Another stated, “There are not enough staff to follow up on screening results properly.”

These findings provide contextual insight into the quantitative results by triangulating quantitative and qualitative evidence. The higher premature mortality observed among men in the quantitative analysis aligns with qualitative findings, where participants consistently identified behavioral risk factors such as smoking and lower healthcare utilization among men as key contributors. Similarly, persistent urban–rural disparities are associated with reported barriers to healthcare access, workforce shortages, and differences in service availability described by participants. This integration suggests that structural and behavioral factors may help explain the observed disparities, although it remains exploratory rather than causal.

Similarly, the persistence of urban–rural disparities corresponds with reported barriers in healthcare access, workforce shortages, and differences in service availability described by participants. These integrated findings highlight how structural and behavioral factors may contribute to the observed patterns in premature mortality, while also underscoring the gap between data availability and its effective use in health system planning.

## Discussion

This study identified a substantial burden of premature mortality from NCDs in Aktobe Region, particularly from CVD and cancer, with consistently higher rates among men and urban populations. The magnitude of these disparities suggests persistent structural and behavioral inequalities in health outcomes.

Higher mortality among men may be linked to greater exposure to behavioral risk factors such as smoking, alcohol consumption, and lower healthcare utilization, while higher urban mortality may reflect a combination of lifestyle factors, environmental exposures, and differences in access to specialized care (15–17). However, given the descriptive nature of the data, these explanations should be considered plausible rather than causal. Although supported by qualitative findings, they remain speculative and cannot be confirmed within the scope of the current study.

The observed increase in premature mortality during the COVID-19 period, particularly for respiratory diseases, may reflect both direct and indirect effects of the pandemic. Disruptions in healthcare services, delayed diagnoses, and increased vulnerability among individuals with pre-existing conditions may have contributed to this pattern, as reported in other settings, including the United States (18, 19). At the same time, the inclusion of acute respiratory conditions within the ICD-10 codes J00–J99 and the potential misclassification of causes of death during the pandemic limit the ability to distinguish between COVID-19-related mortality and longer-term NCD trends. These findings should therefore be interpreted with caution.

Encouragingly, a statistically significant decline in premature mortality from cancer, and reductions in diabetes-related mortality among men, suggest potential improvements in early detection and disease management. These trends may reflect the impact of screening programs and health system interventions implemented in Kazakhstan. However, the absence of adjustment for confounding factors and the short study period limit the ability to attribute these changes to specific interventions. Moreover, several outcomes, including overall premature mortality and respiratory disease mortality, did not show statistically significant trends, indicating that observed fluctuations may reflect random variation rather than sustained population-level changes.

The integration of qualitative and quantitative findings provides additional insight into the mechanisms underlying these patterns. A triangulation approach guided interpretation, whereby convergence between statistical patterns and qualitative themes enhanced interpretation and improved methodological clarity. While quantitative results demonstrate persistent disparities by sex and place of residence, qualitative findings highlight behavioral risk factors, gaps in preventive care, and limitations in the use of epidemiological data for decision-making. Healthcare managers reported that although data are routinely collected, their use for strategic planning remains limited. These disconnects between data availability and effective utilization may contribute to the persistence of disparities observed in the quantitative analysis. Furthermore, structural barriers such as resource constraints, workforce shortages, and limited intersectoral collaboration may hinder the implementation of targeted interventions.

Compared with existing literature, our findings are broadly consistent with studies from Kazakhstan and other countries showing higher NCD-related mortality among men and urban populations (15–17). However, unlike many studies that rely on age-standardized or analytically adjusted estimates, our results are based on crude descriptive data, which limits direct comparability with international studies. As a result, observed differences may reflect variations in demographic structure as well as underlying risk patterns. Differences in methodology, population structure, and health system organization, including the use of crude rates and the shorter observation period, may also contribute to variations in the magnitude and direction of trends (20, 21). These considerations highlight the importance of cautious and critical comparison rather than direct equivalence. The absence of age-standardization also limits direct comparability with international studies using standardized mortality estimates and should be considered when interpreting differences across settings.

From a health systems perspective, reducing premature mortality requires a multifaceted approach that addresses both behavioral risk factors and systemic challenges. Strengthening primary prevention, improving early detection, and enhancing chronic disease management are essential, particularly for high-risk groups such as men and urban populations. In addition, improving the use of routinely collected data for evidence-based decision-making and fostering intersectoral collaboration are critical for translating data into effective public health action (21, 22). However, given the descriptive design of this study, these implications should be interpreted as indicative rather than prescriptive.

Overall, the findings underscore the importance of integrating epidemiological evidence with health system perspectives to understand better and address premature mortality. At the same time, conclusions regarding underlying mechanisms and the effectiveness of interventions remain limited by the study design, lack of adjustment for confounding factors, and short observation period.

### Limitations

This study has several limitations. First, the quantitative analysis was based on secondary administrative data from a single source, which may be subject to reporting inaccuracies and misclassification, particularly during the COVID-19 period. Additionally, the inclusion of ICD-10 codes J00–J99 for respiratory diseases may have led to overestimation of NCD-related mortality, particularly during the pandemic period.

Second, mortality rates were not age-standardized; therefore, comparisons between groups (e.g., sex and urban/rural residence) may partly reflect differences in underlying population age structures rather than true differences in mortality risk.

Third, the study did not account for important socioeconomic and environmental determinants of health, such as income, education, occupational exposures, and healthcare accessibility, which may confound observed associations. Residual confounding is therefore likely, and observed differences between groups should not be interpreted as causal relationships (12, 23, 24).

Fourth, the qualitative component involved a relatively small sample of healthcare managers (n = 10), and participation was voluntary, which introduced potential selection bias and limited the transferability of the findings. Although thematic saturation was achieved and iterative coding procedures were used to enhance analytical rigor, the sample size may still limit the breadth of perspectives captured. In addition, qualitative findings were based on self-reported experiences and perceptions, which may be subject to reporting bias.

Fifth, the relatively short observation period (2019–2023) limits the ability to assess stable long-term mortality trends. AAPC estimates should therefore be interpreted with caution, particularly where confidence intervals were wide and reflected uncertainty in trend estimates.

Finally, the descriptive mixed-methods design limits causal inference, and no longitudinal follow-up was conducted beyond the study period. Integration of quantitative and qualitative findings was conducted primarily at the interpretation stage using a triangulation approach. It did not include formal joint displays or quantitative–qualitative linkage procedures, which may limit the depth of mixed-method integration.

### Conclusion

This study identified persistent disparities in premature mortality from non-communicable diseases in Aktobe Region, with higher mortality among men and urban populations. Declining trends in cancer- and diabetes-related mortality may indicate improvements in prevention and disease management. Although the findings provide useful evidence for regional health planning, they should be interpreted with caution given the descriptive design and data limitations. Further analytical and longitudinal studies are needed to understand the determinants of premature mortality better and support targeted public health interventions.

## Data Availability

The raw data supporting the conclusions of this article will be made available by the authors, without undue reservation.
