# Supplementary material for: Patterns and determinants of premature mortality from non-communicable diseases in Aktobe region, Kazakhstan: a mixed-methods study
Source: Front Public Health. 2026 Jun 15;14:1813999. doi: 10.3389/fpubh.2026.1813999 (PMC13311102; doi:10.3389/fpubh.2026.1813999)
Supplement: Supplementary file 1 [file Supplementary_file_1.docx]

**Supplementary materials 1.**

| **Group** | **ICD-10 and name of disease** | |
| --- | --- | --- |
| Cancer | C00-C14 | Malignant neoplasms of lip, oral cavity and pharynx |
|  | C15-C26 | Malignant neoplasms of digestive organs |
|  | C30-C39 | Malignant neoplasms of respiratory and intrathoracic organs |
|  | C40-C41 | Malignant neoplasms of bone and articular cartilage |
|  | C43-C44 | Melanoma and other malignant neoplasms of skin |
|  | C45-C49 | Malignant neoplasms of mesothelial and soft tissue |
|  | C50-C50 | Malignant neoplasm of breast |
|  | C51-C58 | Malignant neoplasms of female genital organs |
|  | C60-C63 | Malignant neoplasms of male genital organs |
|  | C64-C68 | Malignant neoplasms of urinary tract |
|  | C69-C72 | Malignant neoplasms of eye, brain and other parts of central nervous system |
|  | C73-C75 | Malignant neoplasms of thyroid and other endocrine glands |
|  | C76-C80 | Malignant neoplasms of ill-defined, secondary and unspecified sites |
|  | C81-C96 | Malignant neoplasms, stated or presumed to be primary, of lymphoid, haematopoietic and related tissue |
|  | C97-C97 | Malignant neoplasms of independent (primary) multiple sites |
| Diseases of the circulatory system | I00-I02 | Acute rheumatic fever |
|  | I05-I09 | Chronic rheumatic heart diseases |
|  | I10-I15 | Hypertensive diseases |
|  | I20-I25 | Ischaemic heart diseases |
|  | I26-I28 | Pulmonary heart disease and diseases of pulmonary circulation |
|  | I30-I52 | Other forms of heart disease |
|  | I60-I69 | Cerebrovascular diseases |
|  | I70-I79 | Diseases of arteries, arterioles and capillaries |
|  | I80-I89 | Diseases of veins, lymphatic vessels and lymph nodes, not elsewhere classified |
|  | I95-I99 | Other and unspecified disorders of the circulatory system |
| Endocrine, nutritional and metabolic diseases | E00-E07 | Disorders of thyroid gland |
|  | E10-E14 | Diabetes mellitus |
|  | E15-E16 | Other disorders of glucose regulation and pancreatic internal secretion |
|  | E20-E35 | Disorders of other endocrine glands |
|  | E40-E46 | Malnutrition |
|  | E50-E64 | Other nutritional deficiencies |
|  | E65-E68 | Obesity and other hyperalimentation |
|  | E70-E90 | Metabolic disorders |
| Diseases of the respiratory system | J00-J06 | Acute upper respiratory infections |
|  | J09-J18 | Influenza and pneumonia |
|  | J20-J22 | Other acute lower respiratory infections |
|  | J30-J39 | Other diseases of upper respiratory tract |
|  | J40-J47 | Chronic lower respiratory diseases |
|  | J60-J70 | Lung diseases due to external agents |
|  | J80-J84 | Other respiratory diseases principally affecting the interstitium |
|  | J85-J86 | Suppurative and necrotic conditions of lower respiratory tract |
|  | J90-J94 | Other diseases of pleura |
|  | J95-J99 | Other diseases of the respiratory system |
| Other disease | H00-H59 | Diseases of the eye and adnexa |
|  | H60-H95 | Diseases of the ear and mastoid process |
|  | D50-D89 | Diseases of the blood and blood-forming organs and certain disorders involving the immune mechanism |
|  | F00-F99 | Mental and behavioural disorders |
|  | G00-G99 | Diseases of the nervous system |
|  | K00-K93 | Diseases of the digestive system |
|  | L00-L99 | Diseases of the skin and subcutaneous tissue |
|  | M00-M99 | Diseases of the musculoskeletal system and connective tissue |
|  | N00-N99 | Diseases of the genitourinary system |
|  | D00-D09 | In situ neoplasms |
|  | D10-D36 | Benign neoplasms |
|  | D37-D48 | Neoplasms of uncertain or unknown behaviour |
